# The clinical value of metabolic indicators in immunoglobulin A nephropathy

**DOI:** 10.3389/fmed.2026.1739735

**Published:** 2026-04-15

**Authors:** Limei Zhao, Tingting Zhang, Xiaoshuang Zhou

**Affiliations:** 1 The Fifth Clinical Medical College of Shanxi Medical University, Taiyuan, Shanxi, China; 2 Department of Nephrology, Shanxi Provincial People’s Hospital, The Fifth Clinical Medical College of Shanxi Medical University, Taiyuan, Shanxi, China; 3 Big Data Center of Kidney Disease, Shanxi Provincial People's Hospital, Taiyuan, Shanxi, China; 4 Shanxi Provincial Key Laboratory of Kidney Disease, Taiyuan, Shanxi, China; 5 Medicinal Basic Research Innovation Center of Chronic Kidney Disease, Ministry of Education, Shanxi Medical University, Taiyuan, Shanxi, China

**Keywords:** metabolism, indicator, IgA nephropathy, clinical value, biomarker

## Abstract

Immunoglobulin A nephropathy (IgAN) is a leading cause of end-stage renal disease (ESRD). There is a critical need for sensitive biomarkers to predict disease progression, as traditional indicators like serum creatinine (Scr) have limitations. This study investigates the association of routine metabolic indicators with renal function, pathology, and hospitalization expenses in IgAN patients. In this cross-sectional study, lactate dehydrogenase (LDH) levels were significantly correlated with mesangial hypercellularity (M) and tubular atrophy/interstitial fibrosis (T) in the MEST-C score in the Oxford classification. Furthermore, LDH levels showed a consistent association with estimated glomerular filtration rate (eGFR) and total hospitalization expenses in unadjusted and multivariable-adjusted analyses. Serum uric acid (SUA) levels also demonstrated a strong relationship with renal function. In lipid metabolism, high-density lipoprotein cholesterol was significantly associated with eGFR after multivariable adjustment, whereas triglycerides (TGs) showed correlations with eGFR only in the unadjusted analysis. Serum total protein (TP) levels exhibited a linear positive correlation with eGFR in the unadjusted analysis and emerged as a robust predictor of M, segmental glomerulosclerosis (S), and T lesions in the Oxford Classification. After false discovery rate correction for multiple comparisons, TP was found to be significantly associated with T lesions, which was the most crucial finding of our study, whereas associations involving LDH and other markers were exploratory. Importantly, S and T lesions were strongly associated with a decline in renal function and M1 lesions were found to respond favorably to corticosteroid therapy, whereas T2 lesions signaled an increased risk of progression to ESRD. These hypothesis-generating findings suggest that the strict control of lipid and SUA levels is essential for patients with IgAN in clinical practice. Elevated LDH and decreased TP levels may alert clinicians to the risks of renal function deterioration and ESRD. Moreover, such patients may respond favorably to corticosteroid therapy, which should be considered within the framework of KDIGO 2025 guidelines based on comprehensive risk assessment.

## Introduction

Immunoglobulin A nephropathy (IgAN) is the most common form of primary glomerulonephritis and a leading cause of end-stage renal disease (ESRD) (1, 2). Approximately 20–40% of patients with IgAN progress to ESRD within 10–20 years after diagnosis (3). The main pathological feature of IgAN is the abnormal proliferation of mesangial cells (MCs), which is a key factor in glomerulosclerosis and renal dysfunction (4, 5). Early identification of the deterioration in renal function facilitates timely intervention and delays disease progression; however, sensitive biomarkers for early prediction of IgAN are yet to be identified. Traditional indicators, such as serum creatinine (Scr), have limitations, including delayed response and insufficient sensitivity, rendering them suboptimal for prognostic prediction and therapeutic evaluation (6). In contrast, molecular biomarkers may offer greater sensitivity and accuracy in assessing the physiological and pathological states in IgAN.

In recent years, the role of metabolic abnormalities in the occurrence and development of IgAN has received increasing attention (7, 8). Studies have shown that lactate dehydrogenase (LDH), Serum total protein (TP), uric acid (SUA), and lipid metabolism indicators are closely related to renal inflammation, oxidative stress, and fibrosis process (9, 10). However, current systematic research on the relationship between these metabolic indicators and renal function, pathological changes, and medical resource utilization (such as hospitalization expenses) in IgAN patients, as well as whether they may become potential biomarkers for evaluating the severity and prognosis of IgAN, is still relatively limited.

To address this knowledge gap, a cross-sectional study was conducted to investigate the associations between routine clinical metabolic indicators (including blood glucose [GLUC], LDH, cholesterol [CHO], triglycerides [TGs], high-density lipoprotein CHO [HDL-C], TP, and SUA) and renal function (estimated glomerular filtration rate [eGFR]), renal pathology (Oxford MEST-C score), and total hospitalization expenses in patients with IgAN. The secondary aims were (1) to develop a nomogram model for predicting eGFR based on metabolic indicators to facilitate the early detection of renal function deterioration and (2) to examine the association of LDH, TP, and SUA with the Oxford Classification of IgAN pathology and to compare their predictive performance against Scr in distinguishing pathological severity, thereby providing a scientific basis for clinical decision-making in the management of IgAN.

## Methods

### Study population and design

**Figure 1:**
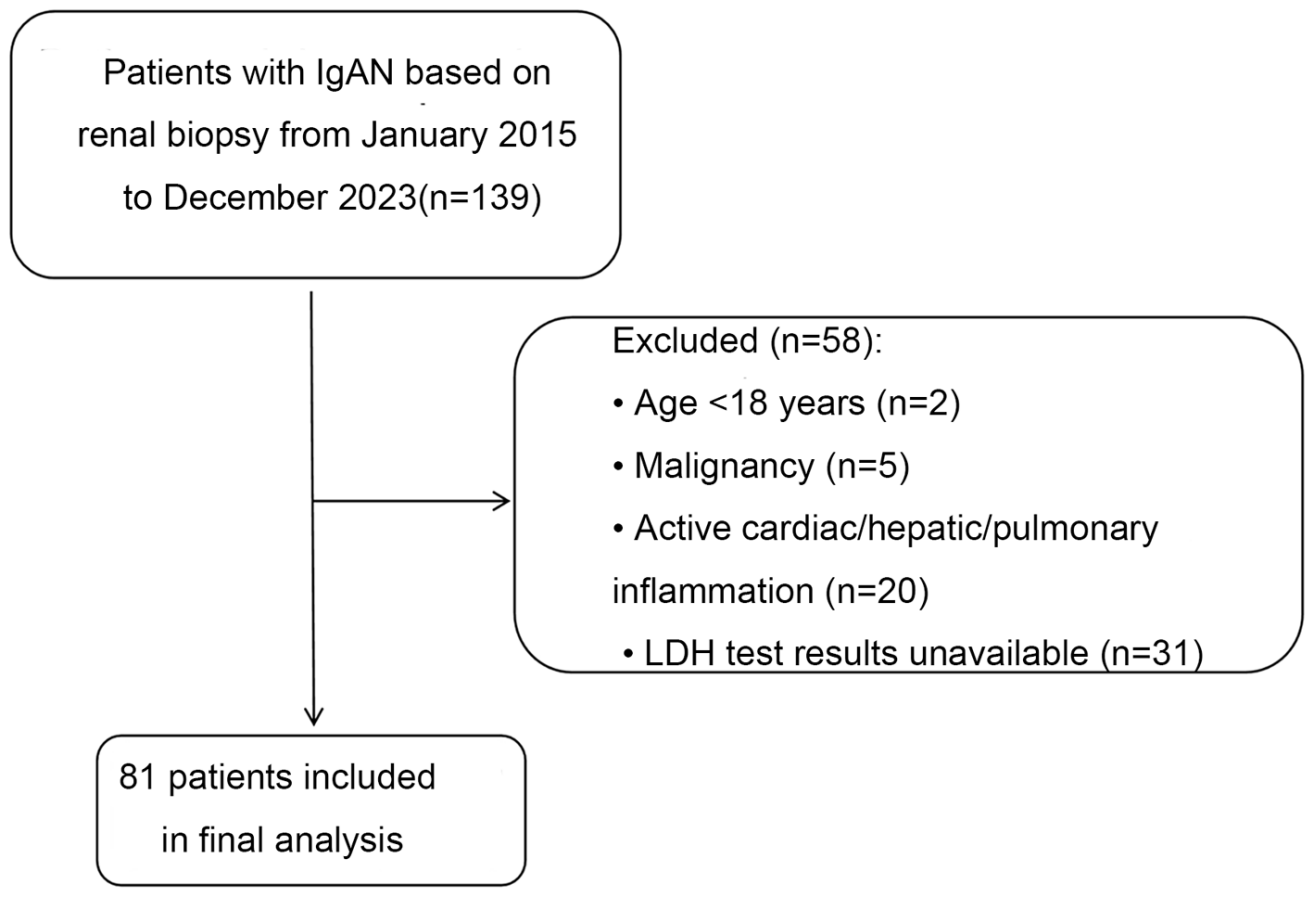
Flowchart.

This cross-sectional study enrolled patients diagnosed with IgAN based on renal biopsy at Shanxi Provincial People’s Hospital between January 2015 and December 2023. The inclusion criteria were (1) biopsy-confirmed IgAN; (2) age ≥18 years; and (3) availability of LDH test results. The exclusion criteria were (1) diagnosis of IgAN at age <18 years; (2) pregnancy; (3) malignancy; and (4) active cardiac, hepatic, or pulmonary inflammation. A total of 81 patients with IgAN were included in this study (Figure 1). The requirement for informed consent was waived owing to the cross-sectional nature of the study and because the database contained no personal patient identifiers.

### Clinical and laboratory data

Demographic, clinical, and laboratory data were collected from the first admission records. Clinical parameters included age, sex, total hospitalization expenses, and hypertension and diabetes history. Laboratory measurements included GLUC, LDH, CHO, TG, HDLC, TP, SUA, urinary total protein (UTP), and Scr. eGFR was calculated using the Chronic Kidney Disease Epidemiology Collaboration equation (11).

### Statistical analysis

Normally distributed variables are expressed as mean ± standard deviation and compared using Student’s *t*-test. Non-normally distributed variables are reported as median (interquartile range) and compared using the Mann–Whitney U test. Categorical variables are presented as numbers (percentages) and analyzed using the Chi-square test. All analyses were performed using R version 4.5.0 (R Foundation for Statistical Computing).

Pearson correlation coefficients and partial correlation coefficients were used to assess relationships between metabolic indicators and both eGFR and total hospitalization expenses in patients with IgAN. A correlation coefficient (*r*) ranging from [−1 to 1] with a *p*-value < 0.05 was considered statistically significant. Factors showing significant correlation with eGFR in patients with IgAN were selected to construct a nomogram prediction model. Quantile–quantile (Q–Q) plots were used to determine whether model residuals conformed to theoretical distributions to evaluate model reliability. Linear regression models were used to assess the predictive capacity of LDH and SCr for total hospitalization expenses, with model assumptions verified using residual plots.

Receiver operating characteristic (ROC) curve analysis was used to evaluate the discriminative performance of metabolic markers (LDH, SCr, SUA, and TP) for each component of the Oxford Classification/MEST-C score (mesangial hypercellularity [M], E, segmental glomerulosclerosis [S], tubular atrophy/interstitial fibrosis [T], and C) in patients with IgAN. The area under the curve (AUC) was calculated with 95% confidence intervals (CIs) using the DeLong method (12). AUC values were interpreted *a priori* as follows: 0.50–0.60 (failed), 0.61–0.70 (poor), 0.71–0.80 (acceptable), 0.81–0.90 (excellent), and 0.91–1.00 (outstanding) (13). The optimal threshold was determined by maximizing the Youden index (sensitivity + specificity – 1). Sensitivity and specificity were reported to inform clinical utility: sensitivity reflects the ability of the marker to correctly identify patients with pathological lesions (rule-in potential), whereas specificity reflects the ability of the marker to correctly identify those without lesions (rule-out potential). To account for multiple comparisons in this exploratory study (4 markers × 5 lesion types), the false discovery rate (FDR) was controlled using the Benjamini-Hochberg method, with statistical significance considered at FDR < 0.05. All ROC analyses were performed using the pROC package in R version 4.5.0.

*Post hoc* power was established and precision analyses were conducted to determine the primary correlations between the metabolic indicators and eGFR. With a sample size of 81 patients and a two-sided significance level of 0.05, this study had 80% power to detect an r value of approximately ≥0.31. Correlations below this threshold may exist but could not be detected, posing a risk of type II error. The 95% CIs for the observed correlations (e.g., LDH with eGFR: *r* = −0.46, 95% CI: −0.618 to −0.271; SUA with eGFR: *r* = −0.42, 95% CI: −0.584 to −0.221) indicated moderate to high precision. However, wider CIs for weaker correlations (e.g., TG with eGFR: *r* = −0.24, 95% CI: −0.449 to −0.024; TP with eGFR: *r* = 0.25, 95% CI: 0.024–0.448) suggest that these estimates should be interpreted with caution.

## Results

### Baseline characteristics of included and excluded patients

This study included 81 eligible patients with IgAN, among whom 29 (35.8%) were female, with a median age of 39 (32–51) years. All participants were of Han Chinese ethnicity. Hypertension was present in 44% of patients, whereas 12% had diabetes mellitus. Significant differences were observed between males and females with respect to SUA, TG, and HDL-C levels (all *p* < 0.05), whereas no significant differences were noted for other measured parameters. The demographic and clinical characteristics of the study population are summarized in Table 1. To assess potential selection bias, the characteristics of included patients were compared with those who were excluded due to missing LDH data or other exclusion criteria (*n* = 58) (Table 2). No significant differences between the groups were noted for any of the measured parameter. These findings suggest that the included patients were representative of the overall IgAN population at our center and that the exclusion of patients lacking LDH data did not introduce substantial selection bias.

**Table 1:** Baseline clinical characteristics of study participants.

Clinical characteristics	Overall (*n* = 81)	Male (*n* = 52, 64.2%)	Female (*n* = 29, 35.8%)	*p*-value
Age (years)	39 (32–51)	38.5 (28.75–51)	39 (32–51)	0.9086
Hypertension, *n* (%)	44 (54%)	30 (57.7%)	14 (48.3%)	0.4147
Diabetes, *n* (%)	10 (12%)	4 (7.7%)	6 (20.7%)	0.08824
GLUC (mmol/L)	5.078 ± 0.591	5 ± 0.608	5.211 ± 0.552	0.1170
LDH (U/L)	199.274 ± 54.097	197.734 ± 55.267	201.806 ± 51.808	0.7417
CHO (mmol/L)	4.759 ± 1.411	4.775 ± 1.62	4.731 ± 0.981	0.8794
TG (mmol/L)	1.867 ± 1.24	2.107 ± 1.442	1.446 ± 0.585	0.0050
HDL-C (mmol/L)	1.132 ± 0.314	1.044 ± 0.292	1.277 ± 0.299	0.0013
TP (g/L)	64.193 ± 7.311	64.2 ± 7.307	64.182 ± 7.447	0.9917
SUA (μmol/L)	396.88 ± 122.578	431.682 ± 118.578	335.678 ± 105.828	0.0004
Scr (μmol/L)	151.097 ± 202.547	175.89 ± 238.343	106.641 ± 102.741	0.0736
UTP (g/24 h)	2.173 ± 2.233	2.405 ± 2.429	1.793 ± 1.859	0.2086

**Table 2:** Comparison of baseline characteristics between included and excluded patients.

Clinical characteristics	Included (*n* = 81)	Excluded (*n* = 58)	*p*-value
Age (years)	39 (32–51)	39 (32–56)	0.657
Female	29 (35.8%)	23 (39.66%)	0.776
Hypertension, *n* (%)	44 (54%)	24 (48.3%)	0.183
Diabetes, *n* (%)	10 (12%)	8 (20.7%)	1.000
GLUC (mmol/L)	5.078 ± 0.591	4.773 ± 0.431	0.193
CHO (mmol/L)	4.759 ± 1.411	4.602 ± 1.594	0.550
TG (mmol/L)	1.867 ± 1.24	1.885 ± 1.133	0.930
HDL-C (mmol/L)	1.132 ± 0.314	1.076 ± 0.338	0.323
TP (g/L)	64.193 ± 7.311	62.559 ± 7.264	0.195
SUA (μmol/L)	396.88 ± 122.578	405.655 ± 123.264	0.679
Scr (μmol/L)	151.097 ± 202.547	166.874 ± 263.427	0.703
eGFR (mL/min/1.73m^2^)	76.068 ± 33.507	77.654 ± 35.268	0.790
UTP (g/24 h)	2.173 ± 2.233	1.964 ± 1.809	0.095

### Correlation analysis between metabolic indicators and eGFR in patients with IgAN

**Figure 2:**
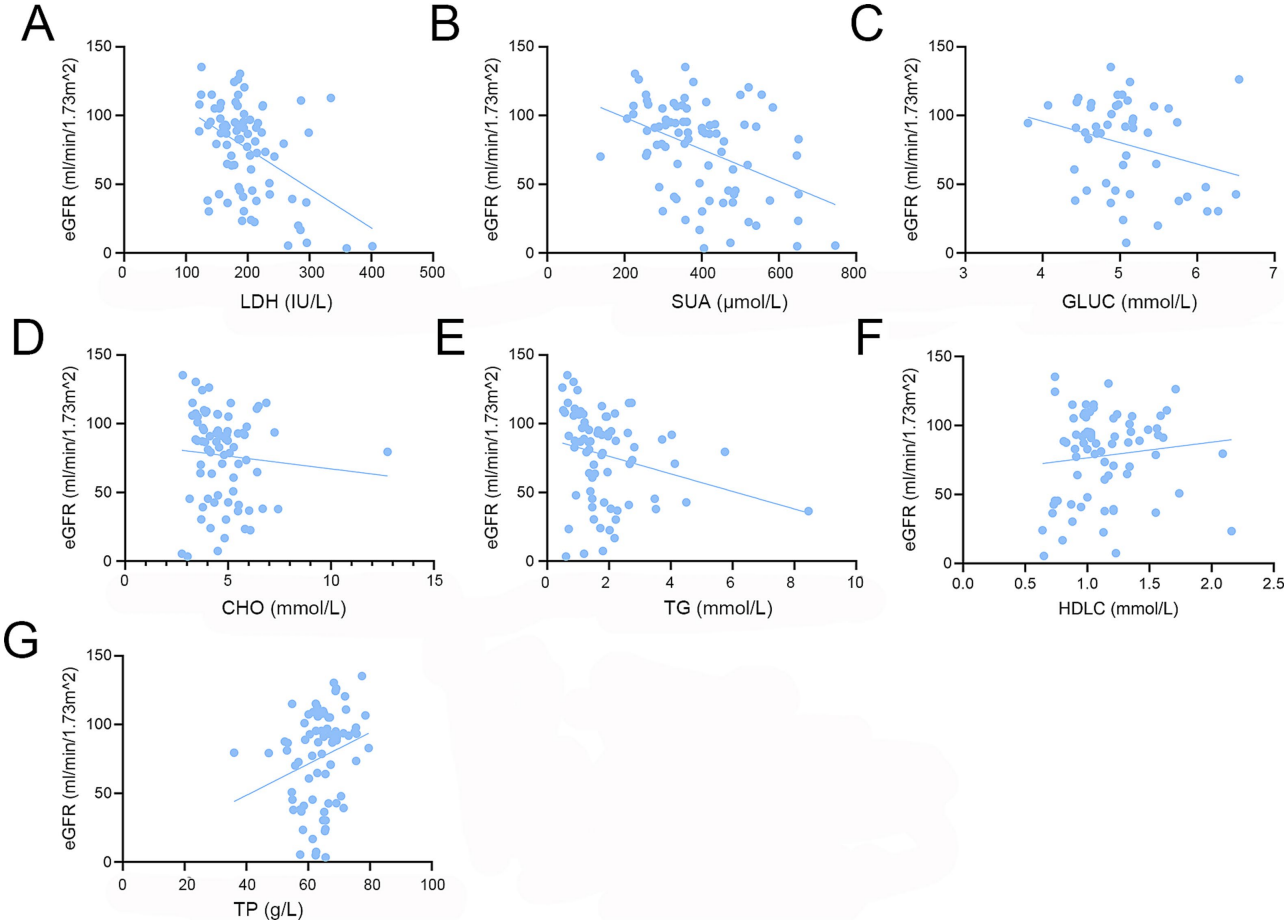
Scatter plots of metabolic indicators versus eGFR in patients with IgAN. **(A)** LDH vs. eGFR; **(B)** SUA vs. eGFR; **(C)** GLUC vs. eGFR; **(D)** CHO vs. eGFR; **(E)** TG vs. eGFR; **(F)** HDLC vs. eGFR; **(G)** TP vs.eGFR.

Pearson correlation analysis revealed significant negative linear correlations between eGFR and LDH, SUA, and TG in patients with IgAN (LDH: *r* = −0.4616, *p* < 0.0001; SUA: *r* = −0.4233, *p* < 0.0001; TG: *r* = −0.2448, *p* = 0.0319), whereas TP exhibited a positive linear correlation with eGFR (*r* = 0.2482, *p* = 0.0307). No significant linear associations were observed between eGFR and GLUC, CHO, or HDLC (Figure 2 and Table 3). The inverse correlations between LDH and SUA with eGFR became more pronounced after adjusting for age, sex, diabetes, UTP, and hypertension (LDH: *r* = −0.5497, *p* < 0.0001; SUA: *r* = −0.4057, *p* = 0.0025); In contrast, the association between TG and eGFR was attenuated and no longer significant after adjustment (*r* = −0.1528, *p* = 0.2748), whereas HDL-C showed a significant positive partial correlation with eGFR (*r* = 0.3876, *p* = 0.0041). The correlation between TP and eGFR also became non-significant after adjustment (*r* = 0.0614, *p* = 0.6625; Table 3).

**Table 3:** Correlation between metabolic indicators and eGFR in IgAN patients.

Indicators	Pearson correlation coefficients		Partial correlation coefficients	
	*r*	*p*	*r*	*p*
LDH	−0.4616	<0.0001	−0.5497	<0.0001
SUA	−0.4233	<0.0001	−0.4057	0.0025
GLUC	−0.2796	0.0517	−0.0167	0.9053
CHO	−0.08111	0.4861	0.1055	0.4523
TG	−0.2448	0.0319	−0.1528	0.2748
HDL-C	0.1123	0.3407	0.3876	0.0041
TP	0.2482	0.0307	0.0614	0.6625

**Figure 3:**
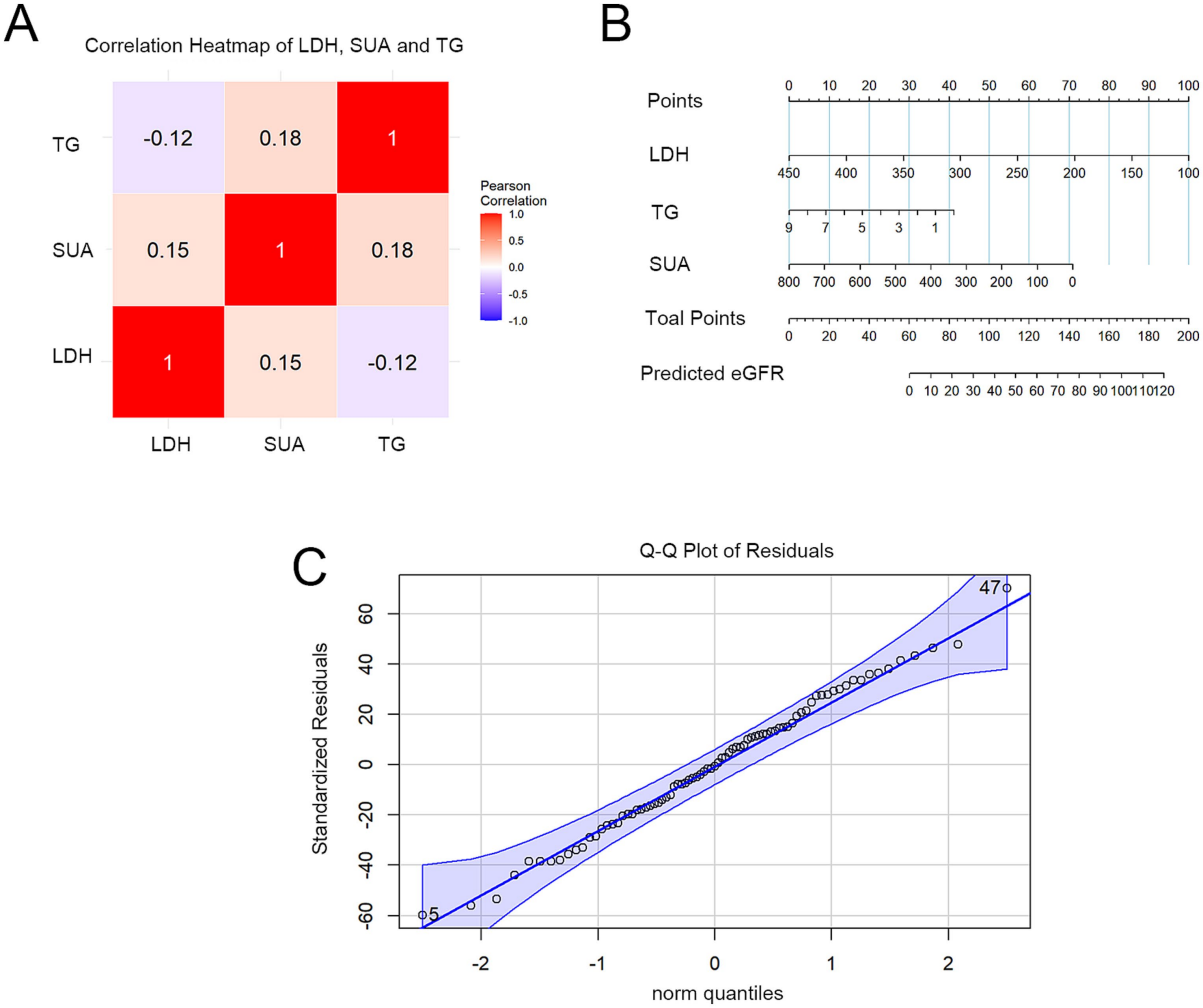
Development of the eGFR prediction nomogram for patients with IgAN. **(A)** Heatmap analysis of correlations among LDH, SUA, and TG; **(B)** eGFR prediction model based on the nomogram; **(C)** Normality assessment of model residuals (Q–Q plot).

Based on Pearson and partial correlation analyses, a predictive model for eGFR was developed for patients with IgAN, incorporating LDH, SUA, TG, HDLC, and TP, and is presented as a nomogram. In the model, individual variable scores are obtained from the vertical axis of the nomogram, and the cumulative total score yields the final risk prediction. Initial model analysis identified LDH and SUA as primary predictors of eGFR; HDLC (*β* = 1.68, *p* = 0.5156) and TP (*β* = 1.06, *p* = 0.5430) showed no significant positive effects; and TG demonstrated a marginally significant negative effect (*β* = −4.51, *p* = 0.0735). Consequently, HDL-C and TP were excluded during model optimization, and a refined nomogram was reconstructed using the remaining variables (Figure 3B). The final nomogram demonstrated both LDH and SUA to have significant negative effects (*p* < 0.05), with the former emerging as the strongest predictor (every 49.31 U/L increase was associated with a 12.27 mL/min/1.73 m^2^ decline in eGFR), followed by SUA (each 168.54 μmol/L increase corresponding to a 15.03 mL/min/1.73 m^2^ reduction) and TG (each 1.00 mmol/L increase leading to a 4.318 mL/min/1.73 m^2^ reduction) (Table 4). Correlation analysis confirmed no significant correlations among LDH, SUA, and TG (*p* > 0.05) (Figure 3A). Analysis of the Q–Q plots demonstrated residuals approximating the diagonal line (Figure 3C), indicating adherence to theoretical distributions. The overall model explained 83% of eGFR variability (*R*^2^ = 0.83, *p* < 0.0001) in the study cohort (Table 4). However, given the relatively small sample size and the absence of an external validation cohort, this model should be considered hypothesis-generating; its predictive performance requires further evaluation in larger, independent datasets before clinical application.

**Table 4:** Effect estimates and variance analysis of the predictive model for eGFR.

Effects						Analysis of variance		
Factor	Low	High	Diff.	Effect	95%CI	Partial SS	Contribution proportion	*p*
LDH	166	214.8	+49.31 U/L	−12.27	[−18.984, −7.588]	16,017.4	49.1%	<0.0001
SUA	306	474.5	+168.54 μmol/L	−15.03	[−22.246,-5.924]	8,774.6	26.9%	0.0010
TG	1.07	2.07	+1.00 mmol/L	−4.318	[−9.216, 0.580]	2,289.1	7.0%	0.0832
REGRESSION						32,608.076	83%	<0.0001

### Correlation between metabolic indicators and Scr with total hospitalization expenses in patients with IgAN

**Figure 4:**
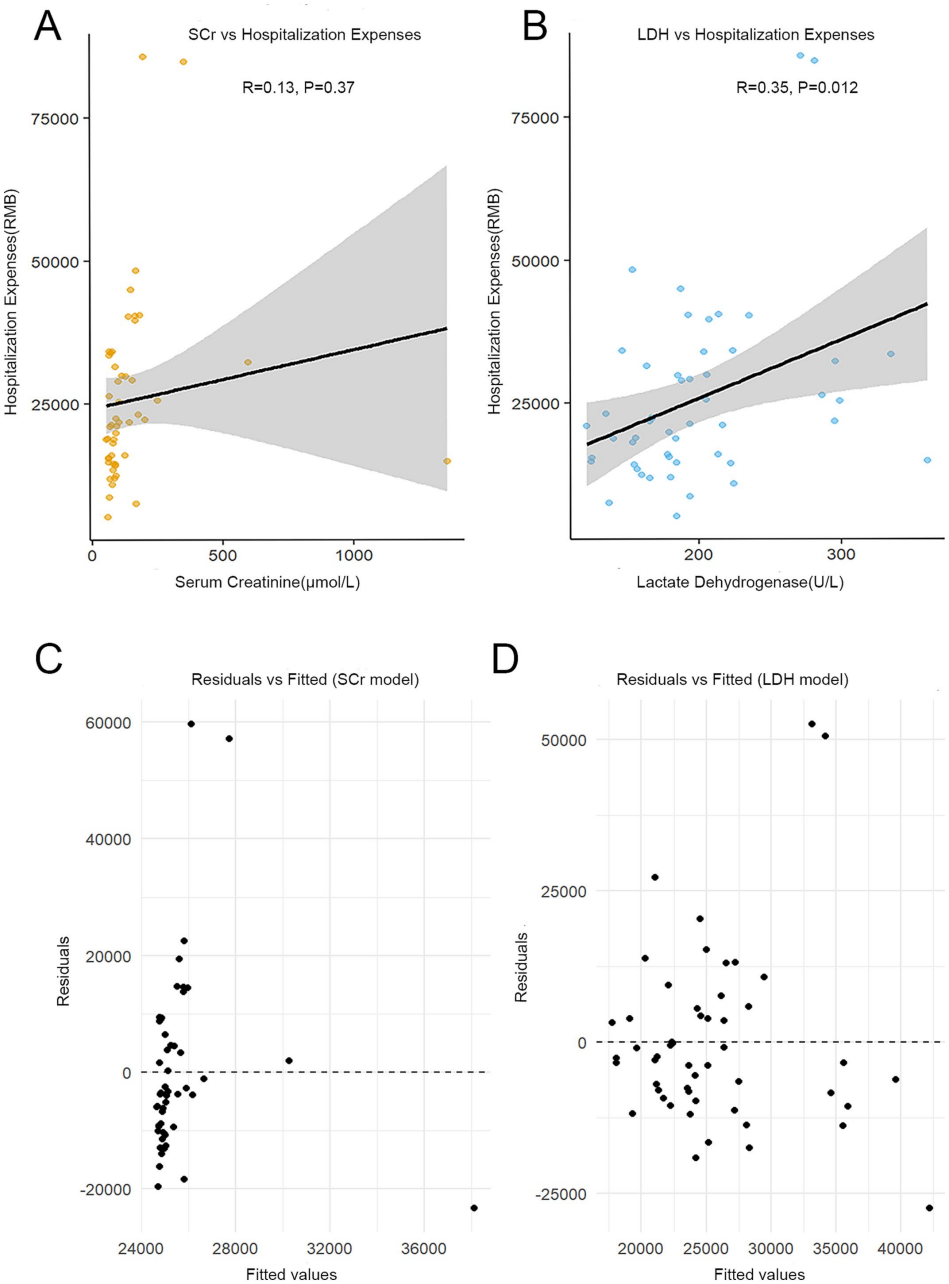
Comparison of the predictive efficacy of LDH and SCr for total hospitalization expenses in patients with IgAN. **(A)** Linear regression model of total hospitalization expenses based on SCr levels; **(B)** Linear regression model of total hospitalization expenses based on LDH levels; **(C)** Residual analysis plot of the SCr prediction model; **(D)** Residual analysis plot of the LDH prediction model.

Pearson correlation analysis revealed a positive correlation between LDH levels and total hospitalization expenses in patients with IgAN (*r* = 0.3512, *p* = 0.0115). This correlation remained statistically significant after adjusting for confounding factors including age, sex, diabetes, UTP, and hypertension (*r* = 0.4239, *p* = 0.0080) (Table 5), suggesting the likely association of LDH with disease severity and progression in IgAN. To further evaluate the clinical utility of LDH in IgAN, the predictive capacity for total hospitalization expenses was compared with that of the conventional renal function marker SCr (Figure 4). LDH exhibited a significant positive correlation with total hospitalization expenses (*p* = 0.012), accounting for approximately 12.3% of the cost variance (*R*^2^ = 0.123). In contrast, SCr showed no significant correlations with total hospitalization expenses (*p* = 0.375), and its explanatory power for total hospitalization expenses was negligible (*R*^2^ = 0.016) (Figures 4A,B). The scatter points were randomly distributed on both sides of *y* = 0 in the residual plots, confirming the robustness of the model (Figures 4C,D). These findings suggest that LDH is a more effective predictor of total hospitalization expenses than SCr in this patient population. However, given the exploratory nature of this analysis and the potential influence of unmeasured confounders (e.g., disease severity at admission, treatment intensity, or socioeconomic factors), this finding should be interpreted with caution and requires validation in future studies.

**Table 5:** Correlation between metabolic indicators and total hospitalization expenses in IgAN patients.

Indicators	Pearson correlation coefficients		Partial correlation coefficients	
	*r*	*p*	*r*	*p*
LDH	0.3512	0.0115	0.4239	0.0080
SUA	0.2647	0.0632	0.1768	0.2884
GLUC	0.1729	0.2559	−0.0614	0.7143
CHO	0.0860	0.5569	−0.3386	0.0576
TG	0.1913	0.1831	−0.2330	0.1593
HDL-C	−0.1604	0.2762	−0.3681	0.0530
TP	0.0684	0.6404	−0.2704	0.1007

### Association between metabolic indicators and renal pathology in patients with IgAN

**Figure 5:**
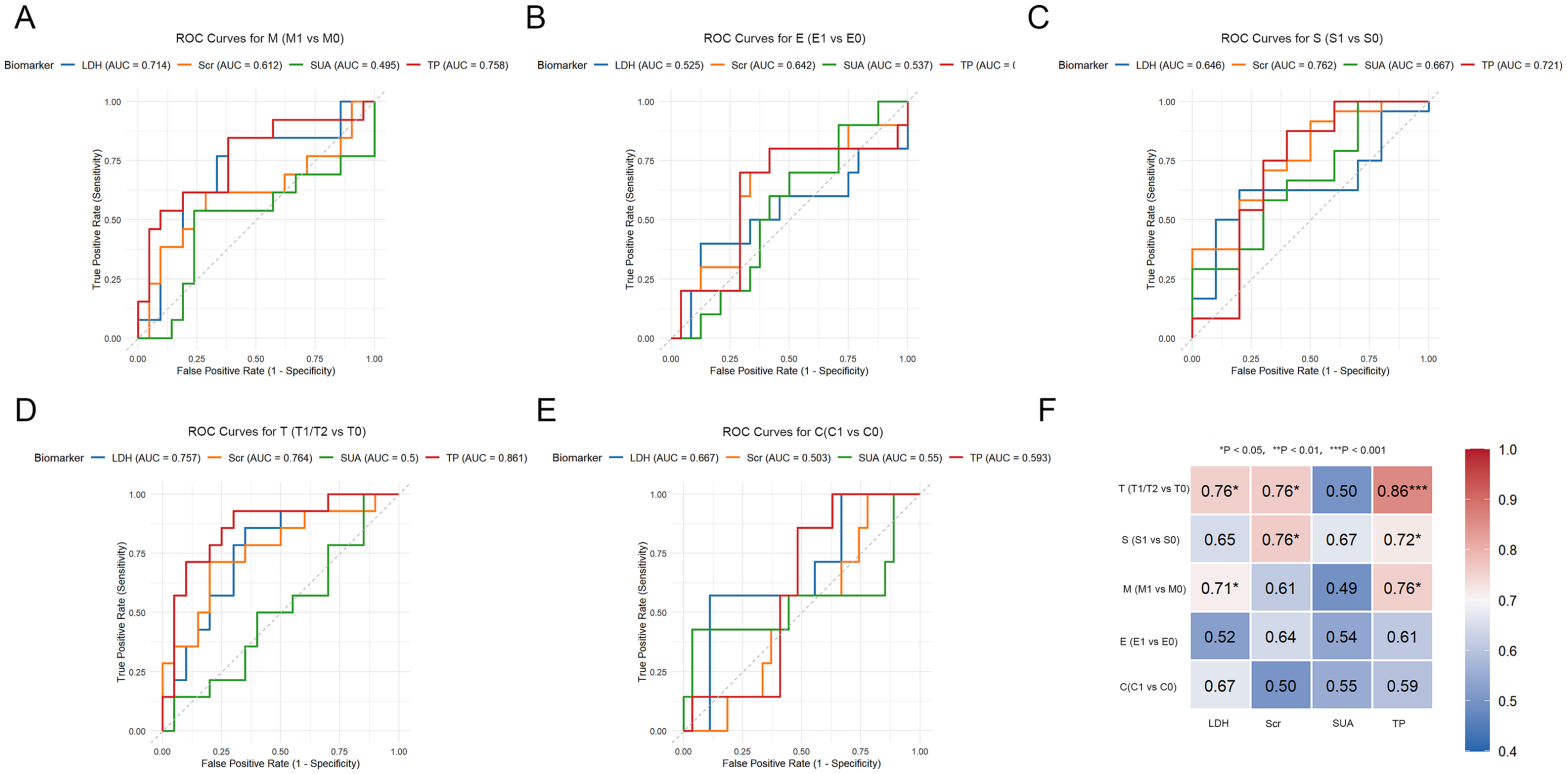
Discriminative performance of LDH, SUA, TP, and SCr for MEST-C scores in IgAN patients (ROC curve analysis). **(A)** ROC curves for M lesions; (B) ROC curves for E lesions; **(C)** ROC curves for S lesions; **(D)** ROC curves for T lesions; **(E)** ROC curves for C lesions; **(F)** Heatmap of AUC values for LDH, Scr, SUA, and TP across MEST-C lesions. *P < 0.05; **P < 0.01; ***P < 0.001.

Lastly, the relationship between metabolic indicators and the Oxford Classification/MEST-C score was evaluated in patients with IgAN. Oxford Classification is a standardized histopathological scoring system used to predict the risk of disease progression and guide treatment decisions (Table 6). Significant differences were noted in LDH (*p* = 0.029) and SUA (*p* = 0.0002) between the T0 vs. T1/T2 groups, whereas other comparisons did not reveal any statistical significance. TP was significantly different in the mesangial hypercellularity (M0 vs. M1/M2; *p* = 0.007), S0 vs. S1 (*p* = 0.045), and T0 vs. T1/T2 groups (*p* = 0.0002; Table 7). To further assess the diagnostic value of these metabolic indicators in IgAN pathology, the discriminative ability of LDH, SUA, and TP was compared versus the conventional marker SCr for Oxford Classification lesions (Figure 5 and Table 8). For M1 vs. M0, both LDH (area under the curve [AUC] = 0.714, 95% CI: 0.58–0.85, sensitivity = 84.6%, specificity = 61.9%, *p* = 0.040) and TP (AUC = 0.758, 95% CI: 0.63–0.89, sensitivity = 84.6%, specificity = 61.9%, *p* = 0.012) demonstrated acceptable discriminative performance, with high sensitivity indicating good rule-in potential for M1 lesions. However, neither reached statistical significance (FDR = 0.18 for LDH and 0.19 for TP) after FDR corrections for multiple comparisons, suggesting that these findings should be interpreted with caution. SCr (AUC = 0.612, 95% CI: 0.46–0.76, *p* = 0.288) and SUA (AUC = 0.495, 95% CI: 0.35–0.64, *p* = 0.972) showed poor discrimination performance. All markers exhibited poor discrimination (AUC < 0.7, all *p* > 0.05, all FDR > 0.05) with respect to endocapillary hypercellularity (E0 vs. E1) and crescents (C1 vs. C0), indicating limited utility for detecting these lesions. SCr showed acceptable discriminative ability for segmental glomerulosclerosis (S0 vs. S1; AUC = 0.762, 95% CI: 0.62–0.90, sensitivity = 91.7%, specificity = 50%, *p* = 0.018), followed by TP (AUC = 0.721, 95% CI: 0.58–0.86, sensitivity = 87.5%, specificity = 60%, *p* = 0.046). Both markers demonstrated high sensitivity (rule-in potential for S lesions) but moderate specificity. Neither association remained significant (FDR = 0.12 for SCr; FDR = 0.19 for TP) after FDR corrections, indicating these findings to be exploratory. TP achieved excellent discriminative performance in assessing tubular atrophy/interstitial fibrosis (T1/T2 vs. T0; AUC = 0.861, 95% CI: 0.77–0.95, sensitivity = 92.9%, specificity = 70%, *p* < 0.001), with high sensitivity indicating strong rule-in potential for T lesions. SCr (AUC = 0.764, 95% CI: 0.64–0.89, sensitivity = 71.4%, specificity = 80%, *p* = 0.010) and LDH (AUC = 0.757, 95% CI: 0.64–0.87, sensitivity = 85.7%, specificity = 65%, *p* = 0.012) showed acceptable discrimination. SUA remained noninformative (AUC = 0.5, 95% CI: 0.35–0.65, *p* = 1.0). Notably, TP remained significantly associated with T lesions after FDR corrections (FDR < 0.001), supporting its robustness as a predictor for tubular atrophy/interstitial fibrosis. Based on clinical consensus, S and T lesions correlate strongly with renal function decline (14, 15), whereas C lesions require aggressive immunosuppression (16). M1/E1 lesions may respond well to corticosteroid therapy (17), whereas T2/C1 lesions indicate a high risk of progression to ESRD. Therefore, for patients with IgAN who exhibit elevated SCr and LDH alongside decreased TP, clinicians must be alert to the risks of renal function deterioration and progression to ESRD.

**Table 6:** MEST-C score.

Scoring item	Score	
Mesangial Hypercellularity (M)	M0	Mesangial hypercellularity in <50% of glomeruli
	M1	Mesangial hypercellularity in ≥50% of glomeruli
Endocapillary Hyperplasia (E)	E0	No endocapillary hypercellularity.
	E1	Endocapillary hypercellularity present in some or all glomeruli
Segmental Glomerulosclerosis (S)	S0	No segmental sclerosis or adhesion
	S1	Segmental sclerosis or adhesion in ≥1 glomerulus
Tubular Atrophy/Interstitial Fibrosis (T)	T0	≤25% cortical area involvement
	T1	26–50% involvement
	T2	>50% involvement
Crescents (C)	C0	No crescents or <10% of glomeruli with cellular/fibrocellular crescents
	C1	≥10% of glomeruli with crescents

**Table 7:** Correlation between metabolic indicators and renal pathology in IgAN patients.

Indicators	M0 (23)	M1 (13)	*p*-value
LDH	186.52 ± 44.24	205.4 ± 45.56	0.239
SUA	416.35 ± 97.8	440.41 ± 136	0.581
HDL-C	0.96 ± 0.15	1.17 ± 0.37	0.070
TG	1.83 ± 1.03	2.34 ± 1.52	0.253
TP	67.81 ± 5.98	60.2 ± 10.58	0.007
	E0 (26)	E1 (10)	
LDH	191.08 ± 38.02	198.96 ± 64.98	0.725
SUA	419.92 ± 117.05	405.79 ± 91.65	0.706
HDL-C	1.01 ± 0.17	0.96 ± 0.22	0.528
TG	2.15 ± 1.39	1.8 ± 0.89	0.412
TP	65.38 ± 8.59	63.75 ± 9.47	0.612
	S0 (10)	S1 (26)	
LDH	176.19 ± 31.42	196.87 ± 47.5	0.142
SUA	371.82 ± 98.58	446.97 ± 117.7	0.067
HDL-C	1 ± 0.21	1.06 ± 0.32	0.517
TG	1.55 ± 0.88	2.23 ± 1.34	0.098
TP	69.43 ± 9.03	63.02 ± 8.07	0.045
	T0 (20)	T1/T2 (16)	
LDH	178.26 ± 35.72	212.19 ± 49.3	0.029
SUA	415.49 ± 115.51	436.93 ± 110.57	0.0002
HDL-C	1.01 ± 0.21	1.08 ± 0.34	0.478
TG	1.92 ± 1.23	2.19 ± 1.31	0.521
TP	68.99 ± 6.01	59.06 ± 8.88	0.0002
	C0 (28)	C1 (8)	
LDH	195.1 ± 45.72	187.17 ± 44.88	0.669
SUA	398.43 ± 88.74	466.79 ± 156.41	0.270
HDL-C	1 ± 0.17	1.04 ± 0.23	0.658
TG	2.04 ± 1.23	2.04 ± 1.42	0.910
TP	65.39 ± 9.15	63.01 ± 7.27	0.492

**Table 8:** Diagnostic performance metrics of LDH, SUA, TP, and SCr in differentiating MEST-C scores in IgAN patients.

Outcome	Biomarker	AUC	Threshold	Sensitivity	Specificity	*p* value
M (M1 vs. M0)	LDH	0.714	187.665	0.846	0.619	0.040
	Scr	0.612	101.390	0.615	0.714	0.288
	SUA	0.495	356.785	0.538	0.762	0.972
	TP	0.758	67.515	0.846	0.619	0.012
E (E1 vs. E0)	LDH	0.525	158.260	0.400	0.875	0.835
	Scr	0.642	98.840	0.800	0.583	0.205
	SUA	0.538	414.295	0.700	0.500	0.752
	TP	0.608	64.070	0.700	0.708	0.341
S (S1 vs. S0)	LDH	0.646	189.530	0.625	0.800	0.192
	Scr	0.762	72.845	0.917	0.500	0.018
	SUA	0.667	293.255	1.000	0.300	0.137
	TP	0.721	69.235	0.875	0.600	0.046
T (T1/T2 vs. T0)	LDH	0.757	187.665	0.857	0.650	0.012
	Scr	0.764	101.390	0.714	0.800	0.010
	SUA	0.500	293.255	1.000	0.150	1.000
	TP	0.861	67.515	0.929	0.700	0.000
C (C1 vs. C0)	LDH	0.667	158.260	0.571	0.889	0.187
	Scr	0.503	174.305	1.000	0.222	1.000
	SUA	0.550	562.615	0.429	0.963	0.708
	TP	0.593	67.515	0.857	0.519	0.478

## Discussion

IgAN is the most common type of primary glomerulonephritis, with a complex pathogenesis influenced by multiple factors (17). LDH, a key enzyme in the intracellular glycolytic pathway, is released into the bloodstream under conditions of tissue injury, inflammation, and hypoxia, and therefore often serves as a non-specific marker elevated in various diseases (18, 19). Pearson correlation analysis revealed LDH levels to be significantly negatively correlated with eGFR and positively correlated with total hospitalization expenses in patients with IgAN; these associations remained significant after adjusting for age, sex, diabetes, UTP, and hypertension. Furthermore, a nomogram prediction model identified LDH as a major predictor of eGFR, with every 49.31 U/L increase in LDH associated with a 12.27 mL/min/1.73 m^2^ decrease in eGFR. With respect to histopathological correlations, ROC analysis demonstrated LDH to have acceptable discriminative ability for mesangial hypercellularity (M1 vs. M0) and tubular atrophy/interstitial fibrosis (T1/T2 vs. T0). However, after FDR correction for multiple comparisons, only the association with T lesions was statistically significant, suggesting that the relationship between LDH and M lesions should be considered exploratory and highlighting the requirement for further validation. Notably, T lesions are strongly associated with a decline in renal function, whereas M1 lesions may respond favorably to corticosteroid therapy. These findings suggest that elevated LDH levels may alert clinicians to monitor patients with IgAN for a potential decline in renal function. However, as emphasized in the KDIGO 2025 guidelines, corticosteroid therapy is recommended for patients at high risk of progression (proteinuria ≥ 0.5 g/day despite optimized supportive care) after careful risk–benefit assessment (20), rather than being reliant solely on biomarker levels. Therefore, whether LDH can refine patient selection for immunotherapy warrants further investigation in prospective studies.

Additionally, abnormal lipid metabolism is noted in the renal tubular cells of patients with IgAN. This dysregulation leads to lipid accumulation, resulting in lipotoxicity and the promotion of TG and CHO synthesis, thereby accelerating tubular injury and atrophy (21, 22). HDL-C may mitigate cellular damage by facilitating reverse CHO transport from the peripheral tissues to the liver (23). Our findings also revealed a linear negative correlation between TG and eGFR in patients with IgAN. However, this correlation became nonsignificant after adjusting for age, sex, diabetes, UTP, and hypertension, whereas HDL-C demonstrated a linear positive association with eGFR. TG demonstrated a marginally significant negative predictive effect on eGFR in the nomogram prediction model for eGFR in patients with IgAN. These findings suggest that lipid metabolism is differentially associated with renal function in IgAN, with HDL-C showing an independent protective association, whereas the relationship between TG and eGFR appears to be influenced by other clinical factors.

Epidemiological data indicate that approximately 25.4% of patients with IgAN have concomitant hyperuricemia. Furthermore, SUA levels have been reported to be closely associated with disease progression and prognosis (24, 25). Similarly, our findings demonstrated a significant correlation between SUA and eGFR in patients with IgAN. The nomogram prediction model revealed that for every 168.54 μmol/L increase in SUA, the eGFR decreased by 15.03 mL/min/1.73 m^2^ in these patients. The underlying mechanisms may involve persistent hyperuricemia, which contributes to the development of IgAN by inducing renal arteriolosclerosis, glomerulosclerosis, and tubulopathy (26, 27). The resulting renal impairment leads to a reduction in uric acid excretion, exacerbating hyperuricemia (28, 29). Elevated SUA subsequently further aggravates renal injury through vascular smooth muscle cell proliferation and endothelial dysfunction, thereby commencing a vicious cycle (30, 31). These findings suggest that strict control of both lipid and uric acid levels in patients with IgAN may help delay the progression of renal function deterioration.

The principal component of TP is serum albumin, which serves as an indicator of the inflammatory microenvironment (32). Elevated levels of inflammatory cytokines and oxidative stress during inflammation can suppress hepatic albumin synthesis (33). Moreover, serum albumin levels are influenced by proteinuria. Studies have demonstrated the antioxidant properties of albumin in mesangial cells, where extracellular albumin attenuates hydrogen peroxide–induced production of reactive oxygen species (34). Thus, serum albumin not only reflects systemic inflammatory status and the severity of proteinuria but also exhibits protective effects against IgAN progression. Pearson correlation analysis revealed a linear positive correlation between TP and eGFR in patients with IgAN. Furthermore, TP demonstrated acceptable to excellent discriminative ability for multiple Oxford Classification lesions, including M1 and S1, and most notably for T1/T2. Importantly, after FDR correction for multiple comparisons, TP continued to be significantly associated with T lesions (FDR < 0.001), representing the most robust finding in our study. Regarding the potential mechanisms underlying the association between TP and T lesions, several aspects can be considered. First, albumin has antioxidant properties; its reduced levels may impair the body’s capacity to clear reactive oxygen species, thereby exacerbating oxidative stress-induced tubular injury (34). Second, hypoalbuminemia often reflects the presence of substantial proteinuria, and filtered proteins can exert direct cytotoxic effects on proximal tubular epithelial cells, promoting inflammatory responses and activating fibrotic pathway (35). Third, albumin binds and transports various bioactive molecules, including inflammatory mediators; decreased albumin levels may thus facilitate local inflammatory responses in the tubulointerstitium (36). Regarding the mechanisms linking low TP levels with M and S lesions, lipid metabolism disorders may be involved. Hypoalbuminemia often stimulates compensatory hepatic lipoprotein synthesis while simultaneously reducing lipoprotein clearance, leading to hyperlipidemia. Hyperlipidemia promotes the generation of oxidized low-density lipoprotein (ox-LDL); ox-LDL is taken up by mesangial cells via scavenger receptors, stimulating mesangial cell proliferation (M lesion) (37). Concurrently, mesangial cells laden with ox-LDL release profibrotic factors such as transforming growth factor-beta (TGF-β) and platelet-derived growth factor (PDGF), accelerating extracellular matrix deposition, which over time can lead to segmental glomerulosclerosis (S lesion) (38–40). These findings suggest low TP levels as a clinically useful indicator for identifying patients with IgAN who are at risk for tubular atrophy/interstitial fibrosis, which strongly correlates with renal function decline. Close monitoring of renal function is warranted for patients with IgAN having reduced TP levels, as low TP may identify individuals at higher risk for disease progression. The KDIGO 2025 guidelines emphasize that treatment decisions should be guided by a comprehensive risk assessment, including proteinuria, eGFR, and histological findings (20), rather than relying on individual biomarkers in isolation. The robust association between TP and T lesions observed in our study suggests that TP may complement existing risk-stratification tools; however, its role in guiding therapeutic decisions needs to be validated in larger, prospective cohorts.

The differential association patterns of LDH and TP with Oxford lesions provide insights into their distinct biological roles. LDH, as an intracellular enzyme released during cell injury or necrosis, predominantly reflects tubular damage, which may explain its selective association with T lesions. In contrast, TP is a multifaceted biomarker integrating information on nutritional status, systemic inflammation, and lipid metabolism. Its broader associations with M, S, and T lesions suggest that TP captures not only tubular injury but also glomerular pathological processes, including mesangial proliferation and segmental sclerosis. These findings imply that while LDH may serve as a marker of tubular damage, TP represents a more global indicator of disease severity in IgAN, reflecting the interplay between systemic derangements and diverse renal pathological compartments.

The current study has several limitations that should be considered when interpreting our findings. First, the eGFR prediction nomogram achieved a high *R*^2^ value (0.83) in the study cohort, but this was derived from a relatively small sample (*n* = 81) without internal or external validation. Thus, the model may be susceptible to overfitting, and its predictive performance should be validated in larger, independent cohorts. Second, the generalizability of our results should be interpreted within the context of the study population: the single-center cohort consisting of exclusively Han Chinese participants (44% hypertension, 12% diabetes), which aligns with previously reported IgAN populations in China (41) but may differ from cohorts in other regions. Given the potential variations in genetic background, environmental factors, and metabolic profiles across populations, caution should be exercised when extrapolating our findings to other ethnic or geographic groups. Third, while this study included patients with varying degrees of proteinuria, the small sample size did not permit subgroup analysis stratified by proteinuria status. Whether the observed associations hold true for patients with IgAN with proteinuria vs. those without proteinuria is yet to be determined. Fourth, medication use and systemic inflammatory markers were not accounted for in this study, and although patients with active cardiac, hepatic, or pulmonary inflammation were excluded, residual confounding from these factors cannot be excluded, warranting validation in future prospective studies.

Notwithstanding these limitations, our findings align with the research priorities outlined in the KDIGO 2025 guidelines, which highlight the need for validating the International IgAN Prediction Tool in diverse populations and the urgent need to identify biomarkers for the prognosis, treatment selection, and monitoring of therapeutic response (20). The associations observed between LDH, TP, and Oxford MEST-C lesions, particularly the robust relationship between TP and T lesions after multiple comparison correction, suggest that these routine metabolic indicators may serve as readily accessible biomarkers for risk stratification. However, given the cross-sectional, single-center nature of our study, prospective validation using larger and more diverse multicenter cohorts is essential before these markers can be integrated into clinical practice. Future studies should determine whether incorporating LDH and TP into existing prediction tools can improve dynamic risk assessment and guide treatment decisions, particularly in distinguishing patients who may benefit from immunotherapy versus those requiring intensification of supportive care alone.

## Conclusion

The metabolic indicators LDH, SUA, TG, HDL-C, and TP demonstrated significant correlations with eGFR levels in patients with IgAN. LDH and SUA, specifically, were primary negative predictors for eGFR in these patients. Furthermore, elevated LDH levels exhibited a significant positive correlation with hospitalization expenses. Patients with IgAN presenting with concurrent elevation of LDH and reduction in TP exhibited a greater propensity for renal pathological progression to the Oxford Classification stages M1, S1, and T1/T2. These findings indicate LDH and TP to be closely associated with disease progression and clinical prognosis in IgAN. Consequently, the stringent management of serum lipid and SUA levels is essential for patients with IgAN in clinical practice. Moreover, increased vigilance is warranted regarding the risk of renal function deterioration and progression to ESRD for patients presenting with elevated LDH levels accompanied by decreased TP. Glucocorticoid therapy may be considered in such cases within the framework of the KDIGO 2025 guidelines based on comprehensive risk assessment.

## Data Availability

The original contributions presented in the study are included in the article/supplementary material, further inquiries can be directed to the corresponding author.
